# Central Corneal Thickness of a Saudi Population in Relation to Age, Gender, Refractive Errors, and Corneal Curvature

**DOI:** 10.7759/cureus.30441

**Published:** 2022-10-18

**Authors:** Abdulrahman A Almazrou, Wala A Abualnaja, Amani A Abualnaja, Ahmed Z Alkhars

**Affiliations:** 1 Ophthalmology, Imam Mohammad Ibn Saud Islamic University, Riyadh, SAU; 2 Optometry, Ohud Hospital, Riyadh, SAU; 3 General Physician, Imam Mohammad Ibn Saud Islamic University, Riyadh, SAU; 4 Pediatrics, College of Medicine, King Faisal University, Al-Ahsa, SAU

**Keywords:** astigmatism, myopia, refractive error, corneal curvature, central corneal thickness

## Abstract

Background

In this study, we aimed to investigate the relationship between central corneal thickness (CCT) and age, gender, refractive errors, and corneal curvature in a Saudi population.

Methodology

In this randomized, hospital-based, retrospective study, data were collected from Dr. Sulaiman Al Habib Hospital and Imam Medical Center, Riyadh. A total of 1,005 eyes were included and recruited from patients referred to the refractive surgery clinic for Lasik assessment. The study included patients aged between 17 and 57 years with no history of any ocular pathology, eye surgeries, and systemic disease, as well as all groups with stable refractions. The identifying data were the age and gender of the patients, as well as their CCT, refraction, and corneal curvature. CCT and corneal curvature were measured by ultrasound pachymeter Pentacam.

Results

The distribution of CCT was 543.81 ± 34.47 μm. A significant difference in the mean CCT was observed across different refractive errors (p = 0.004). Patients with astigmatism had the lowest CCT, followed by myopic and hyperopic patients. An association between the spherical equivalent of patients with myopia and CCT (p = 0.001) was noted.

Conclusions

In the Saudi population, we found no significant association between mean corneal curvature and CCT in all three groups. Spherical equivalent in myopic patients was significantly associated with CCT. A significant difference in the mean CCT was observed across different refractive errors. There was a significant negative and weak correlation between age in myopic and astigmatism patients and CCT. Gender was significantly associated with CCT in patients with astigmatism.

## Introduction

The cornea is important for the optical system, and its condition is identified by the nature of vision. Estimation of central corneal thickness (CCT) and endothelial cell parameters is significant when undertaking a useful and morphologic assessment of the cornea for the purpose of diagnosis and endothelial capacity. CCT is a basic parameter in the evaluation and diagnosis of corneal illness [1-3]. Furthermore, with the advancement of corneal refractive surgeries, CCT values are extremely significant during the pre-surgical assessment of the patients as they impact the choice of whether to perform a medical procedure, the type of recommended surgery, and the percentage of complications after the procedure [4-6]. A refractive error is a very common eye issue [7]. It occurs when the eye cannot focus on objects of the outside world, resulting in a blurred image [7]. The consequence of refractive concerns is obscured vision, which in some cases is so severe that it causes visual impairment [7]. In addition, CCT is a measure of corneal rigidity and subsequently affects the precision of intraocular pressure estimation by applanation tonometry [4]. Moreover, CCT is an important parameter in glaucoma [8-10]. Higher CCTs result in artificially higher readings [11]. Lower CCTs influence the measurement of intraocular pressure, underestimating the real value, and, as such, may lead to the progression of primary open-angle glaucoma (POAG) [12,13].

There are several types of refractive errors. The first is myopia wherein patients can clearly see close objects but distant objects are blurry [14]. The second is hyperopia wherein patients can see close objects more blurry than distant objects [15]. The third is astigmatism which is a misshaped cornea or lens which prevents light from focusing properly on the retina [16].

Multiple studies have investigated the correlation between myopia and CCT and have reported conflicting results. One study on the relationship between CCT and the degree of myopia among Saudi adults aged 18-56 years found that there was no correlation between CCT and the degree of myopia [4]. Zhou et al. investigated the distribution of corneal thickness and its relationship with corneal topography in an ametropic population and found a weak significant correlation [17]. Another study discussed the relationships between central and peripheral corneal thickness in different degrees of myopia. It reported no significant differences among low, moderate, and extremely myopic eyes related to the CCT and peripheral corneal thickness. Corneal thickness was very similar in myopic eyes with small differences that were not clinically relevant to myopic patient management [18].

In 2016, Yasir et al. reported that CCT significantly positively correlated with spherical equivalence [19]. Mimouni et al. reported a significant relationship between CCT and myopia [20]. A study conducted in Baghdad by Rashid and Farhood regarding the measurement of CCT by ultrasonic pachymeter and oculus Pentacam® in patients with well-controlled glaucoma showed that there was no significant difference in CCT readings in both glaucoma and control groups [21]. Due to the lack of studies in this field in Saudi Arabia, this study aimed to investigate the relationship between CCT and age, gender, refractive errors, and corneal curvature.

## Materials and methods

Study design

A randomized, hospital-based, retrospective, quantitative study was designed to investigate the relationship between CCT and age, gender, refractive errors, and corneal curvature. This study was conducted from October 2021 to December 2021 at the Imam Medical Center in Riyadh, Saudi Arabia, and Dr. Sulaiman Al Habib Hospital, Takhassusi Street, Riyadh, Saudi Arabia. The study adhered to the tenets of the Declaration of Helsinki 2013. The study was approved by the Institutional Review Board (IRB) at Imam Mohammad Ibn Saud Islamic University (IRB number: 130-2021) in September 2021.

Participants and procedures

A total of 1,005 eyes were included and recruited from patients referred to the refractive surgery clinic for Lasik assessment. The data were collected from Dr. Sulaiman Al Habib Specialist Hospital and Imam Medical Center. The sample size was calculated using the following formula: n = z2pq\d2. The confidence level was determined to be 95%, an estimated proportion of 50% was utilized, a 5% level of precision was set, and an estimated population of 5,368 was used (the population was estimated as follows: the weekly average for operations performed in the first center was eight for a yearly total of 864 operated eyes considering the operation included both eyes, and the weekly average for operations performed in the second center was 42 for a yearly total of 4,536 operated eyes considering the operation included both eyes. This makes the estimated population for both centers 5,368). The minimum sample size was calculated to be 359 eyes.

This study included Saudi adult patients aged between 17 and 57 years with no history of any ocular pathology, no history of eye surgeries, no systemic disease, and all groups with stable refractions. However, this study excluded patients aged less than 17 years; eyes with corneal pathology such as edema, scarring, or corneal dystrophy; eyes with a history of refractive or ocular surgery; history of ocular trauma; and glaucomatous eyes. Patients with systemic disease (such as diabetes or rheumatoid arthritis) and pregnant females were also excluded. According to the sample size, we included all patients assessed for refractive surgery were included in the study.

The identifying data were age and gender, and CCT, refraction, and corneal curvature were assessed. CCT was measured using an ultrasound pachymeter, refraction was measured using an autorefractor and confirmed subjectively by trial lenses and retinoscopy, and refraction was calculated in D as the spherical equivalent which equals to spherical refractive error plus (0.5 × cylindrical refractive errors). The corneal curvature was measured using an auto-refracto-keratometry which measured the two major corneal radii separated by 90° (k1 and k2), and the average of both values was recorded as the average corneal curvature.

Statistical analysis

Data analysis was performed using SPSS version 23 (IBM Corp., Armonk, NY, USA). Frequency and percentages were used to display categorical variables. Mean and standard deviation was used to present continuous variables. Pearson’s correlation was used to test for correlation between continuous variables. Independent t-test and analysis of variance test were also used to test for association. The level of significance was set at 0.05.

## Results

A total of 1,005 eyes were included in this study. Table 1 displays the overall profile of patients. Regarding gender, 389 (38.7%) eyes were of male patients, and 616 (61.3%) eyes were of female patients. In total, 496 (49.9%) of the included eyes were left eyes, and 409 (50.6%) of the included eyes were right eyes. Regarding the center from which the participants were recruited, 882 (87.8%) were recruited from Dr. Sulaiman Al Habib Specialist Hospital, and 123 (12.2%) were recruited from the Imam Medical Center. The mean age of the patients was 29.13 ± 7.04 years. The mean corneal curvature was 42.91 ± 1.47.

**Table 1:** Overall profile of the patients (n = 1,005).

Patients’ profile (n = 1,005)		
Categorical characteristics	n	%
Gender		
Male	389	38.70
Female	616	61.30
Eye		
OS	496	49.40
OD	509	50.60
Center		
Dr. Sulaiman Al-Habib Specialist Hospital	882	87.80
Imam Medical Center	123	12.20
Numerical charactaristics	Mean	Standard deviation
Age	29.13	7.04
Mean corneal curvature	42.91	1.47

**Figure 1:**
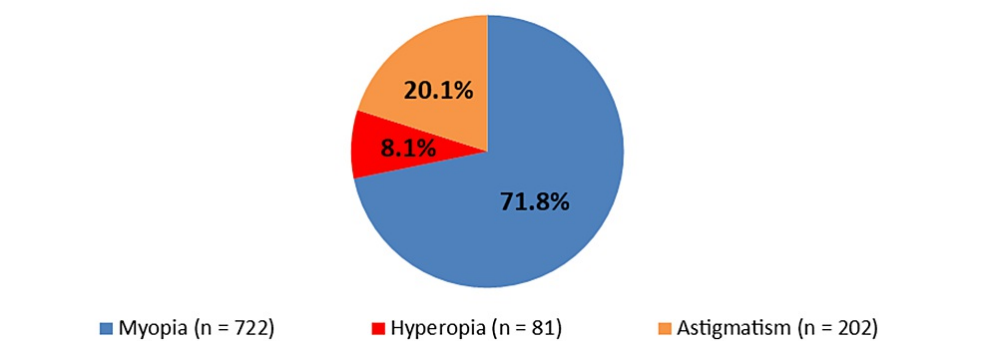
Participants’ refractive error.

**Figure 2:**
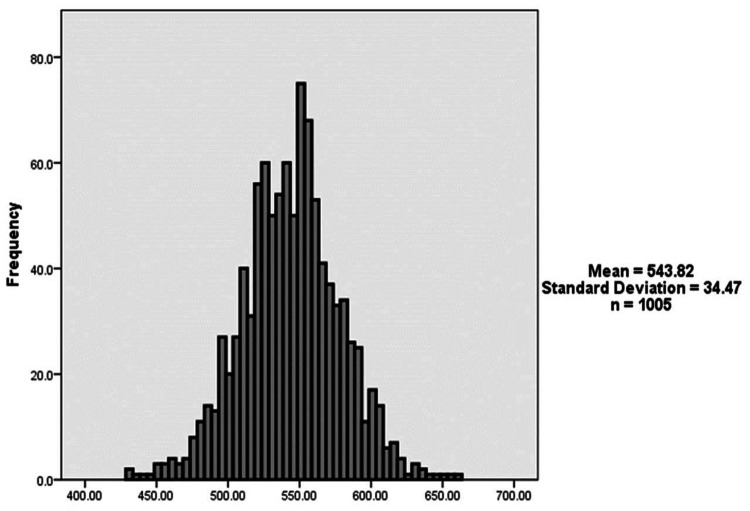
The distribution of central corneal thickness.

Figure 1 shows the participants’ refractive error. A total of 722 (71.8%) patients had myopia, 81 (8.1%) patients had hyperopia, and 202 (20.1%) patients had astigmatism. Figure 2 demonstrates the distribution of CCT. The mean CCT was 543.81 ± 34.47 μm.

Table 2 demonstrates the correlation of factors with CCT within each type of refractive error. Regarding the association testing in patients with myopia, a significant negative weak correlation was noted between age and CCT (p < 0.001, Pearson’s correlation = -0.2). Sphere equivalent was also significantly associated with CCT (p = 0.001, Pearson’s correlation = -0.19). Gender and mean corneal curvature were not significantly associated with CCT. For the association testing in patients with hyperopia, gender, age, sphere equivalent, and mean corneal curvature were not significantly associated with CCT. Regarding the association testing in patients with astigmatism, gender was significantly associated with CCT (p < 0.001), and females were observed to have significantly higher mean CCT compared to males (543.93 ± 33.22 μm vs. 533.83 ± 33.49 μm). A significant negative weak correlation was also noted between age and CCT (p = 0.037, Pearson’s correlation = -0.15). Side, sphere equivalent, mean corneal curvature, astigmatism degree, and axis were not significantly associated with CCT.

**Table 2:** The factors associated with central corneal thickness within each type of refractive error. *Significant at level 0.05.

Factor	Central corneal thickness		P-value
	Mean	Standard deviation	
Association testing in patients with myopia			
Gender			0.758
Male	545.28	35.22	
Female	544.45	34.20	
Eye			0.661
OD	545.32	34.13	
OS	544.19	34.97	
Age with central corneal thickness			
P-value		<0.001*	
Pearson’s correlation		-0.20	
Mean corneal curvature with central corneal thickness			
P-value		0.95	
Pearson’s correlation		0.00	
Sphere equivalent with central corneal thickness			
P-value		0.001*	
Pearson’s correlation		-0.19	
Association testing in patients with hyperopia			
Gender			0.342
Male	543.00	28.67	
Female	550.85	37.52	
Eye			0.814
OD	547.43	34.34	
OS	549.29	35.96	
Age with central corneal thickness			
P-value		0.35	
Pearson’s correlation		-0.11	
Mean corneal curvature with central corneal thickness			
P-value		0.42	
Pearson’s correlation		0.90	
Sphere equivalent with central corneal thickness			
P-value		0.98	
Pearson’s correlation		0.00	
Association testing in patients with astigmatism			
Gender			0.033*
Male	533.8269	33.49	
Female	543.9286	33.22	
Eye			0.956
OD	538.59	34.81	
OS	538.86	32.71	
Age with central corneal thickness			
P-value		0.037*	
Pearson’s correlation		-0.15	
Mean corneal curvature with central corneal thickness			
P-value		0.60	
Pearson’s correlation		-0.04	
Sphere power with central corneal thickness			
P-value		0.60	
Pearson’s correlation		0.04	
Astigmatism with central corneal thickness			
P-value		0.83	
Pearson’s correlation		0.02	
Axis with central corneal thickness			
P-value		0.58	
Pearson’s correlation		-0.04	

Table 3 illustrates the comparison of CCT across refractive error types. A significant difference in the mean CCT was observed across different refractive errors (p = 0.004). Patients with astigmatism had the lowest CCT (538.73 ± 33.66 μm), while patients with hyperopia had the highest CCT (548.23 ± 34.84 μm).

**Table 3:** The comparison of central corneal thickness across refractive error types. *Significant at level 0.05.

Factor	Central corneal thickness		P-value
	Mean	Standard deviation	
Gender			0.004*
Myopia	544.74	34.54	
Hyperopia	548.23	34.84	
Astigmatism	538.73	33.66	

## Discussion

This retrospective study evaluated the CCT in a typical Saudi population and examined the relationship between CCT and age, gender, and refraction. The mean CCT was 543.81 ± 34.47 μm. This is similar to the results reported by Al-Mezaine et al. in which the CCT was evaluated in a Saudi emmetropic population (545.7 ± 27.6 μm) [4]. The measured CCT was similar to that reported in a study of Iraqi citizens (543.95 ± 32.58 μm) [19]. In comparison to neighboring nations, CCT was lower than the Turkish population (552 μm) and the Iranian population (555.6 μm) [22-25]. According to a review of the literature, there may be ethnicity-related differences in CCT globally. In a San Francisco glaucoma clinic, Aghaian et al. [26]. investigated corneal thickness in an ethnically varied group of 801 eyes. All patients had a mean CCT of 542.9 μm. Among all races, African Americans had the lowest CCT (521.0 μm), followed by Hispanics (548.1 μm) and whites (550.4 μm). Japanese participants had the lowest CCT (531.7 μm) among Asian subpopulations compared to Filipino (550.6 μm) and Chinese (555.6 μm) populations.

Our study showed a significant negative weak correlation between age in myopic and astigmatism patients and CCT. According to several studies, CCT decreases significantly with age [19,24,27-29]. Hahn et al. concluded that the decline in keratocyte density with age is responsible for the drop in CCT values [30]. A few studies found no statistically significant relationship between age and CCT. Gross-Otero et al. Identified an association between CCT and age in 375 eyes of a Spanish population. However, the differences are so small that they would be meaningful with a large number of patients [31]. In 109 white European patients, Jonuscheit and Doughty found no age-related variations in CCT (p = 0.381) [32].

Gender was found to be unrelated to CCT. Most of the research that studied the relationship between CCT and gender showed no statistically significant differences [19,29-35].

On the other hand, some studies have reported a statistical link between gender and CCT [24,27,36]. According to Han et al., the difference in CCT between genders was just 4.6 μm, which is less than the mean interocular difference in CCT (7.7 μm). The study reported that the difference in CCT between men and women was statistically significant but not clinically meaningful [11].

Furthermore, there was no statistically significant difference between mean corneal curvature and CCT. This result was consistent with the study by Chen et al. which found no significant relationship between mean corneal curvature and CCT in normal Taiwanese Chinese people aged 40-80 years (Pearson r = 0.013; p = 0.770) [37]. The relationship between CCT and corneal curvature is controversial. Kadhim and Farhood documented a statistically significant (weak) negative correlation between average corneal curvature and CCT (Pearson r =−0.097, p = 0.048) [19]. In the Tajimi Study from Japan, Atsuo et al. discovered a negative association between the two measures [38]. In Japanese individuals aged 40 and above, Sawada et al. found a positive association [39].

This study found a significant difference in the mean CCT across different refractive error types (p = 0.004). Patients with astigmatism had the lowest CCT (538.73 ± 33.66 μm), whereas those with hyperopia had the highest CCT (548.23 ± 34.84 μm). Many researchers have documented the effect of refractive status on CCT, such as Kadhim and Farhood [19], Chang et al. [40], Mohammed et al. [41], and Nemesure et al. [42]. CCT was directly related to refractive error. Other studies reported conflicting findings. According to Price et al., there was no association between refraction and CCT [43,44]. Ortiz et al [18] and Liu and Pflugfelder [45] found no link between CCT and the degree of myopia in corneal thickness in either contact lens wearers or non-wearers. Pederson et al. deduced that there was no statistical difference in CCT between emmetropes and myopes [43].

This study showed that sphere equivalent in myopic patients was significantly associated (p = 0.001) with CCT. Another study in 2016 concerning CCT in the Iraqi population concluded that CCT significantly positively correlated with spherical equivalence [19]. In addition, a study conducted in Brazil in 2017 about the correlation between CCT and myopia found a significant relationship with CCT. These findings contributed to the overall understanding of this study [20]. However, a study done in Saudi Arabia showed no correlation between CCT and the degree of myopia [4]. Kadhim and Farhood [19], Nemesure et al. [42], and Pederson et al. [43] concluded that in myopia degrees, there was no change in CCT.

Limitations

The axial lengths in myopic patients were not measured to differentiate between myopia caused by axial lengths from myopia caused by the cornea. However, some studies have demonstrated no association between CCT and axial length [22,23].

## Conclusions

This study showed a mean CCT of 543.81 + 34.47 μm. We found a significant negative and weak correlation between age in myopic and astigmatism patients and CCT. Gender was not significantly associated with CCT in myopic and hyperopic patients, while in patients with astigmatism, females were observed to have a significantly higher mean CCT compared to males. In addition, there was no significant association between mean corneal curvature and CCT in all three groups. Spherical equivalent in myopic patients was also significantly associated with CCT. A significant difference in the mean CCT was observed across different refractive errors, and patients with astigmatism had the lowest CCT, followed by myopic and hyperopic patients.
